# Zooplankton monitoring to contribute towards addressing global biodiversity conservation challenges

**DOI:** 10.1093/plankt/fby030

**Published:** 2018-08-25

**Authors:** Sanae Chiba, Sonia Batten, Corinne S Martin, Sarah Ivory, Patricia Miloslavich, Lauren V Weatherdon

**Affiliations:** 1 JAMSTEC, 3173-25 Showamachi, Kanazawaku, Yokohama, Japan; 2 UN Environment World Conservation Monitoring Centre, 219 Huntingdon Road, Cambridge, UK; 3 Marine Biological Association, c/o 4737 Vista View Cr, Nanaimo BC, Canada; 4 University of Tasmania, Private Bag 110, Hobart TAS, Australia; 5 Australian Institute of Marine Science, PMB No 3, Townsville MC, QLD, Australia; 6 Universidad Simon Bolivar, Valle de Sartenejas, Caracas, Venezuela

**Keywords:** zooplankton, monitoring, indicators, Aichi Biodiversity Targets, EOVs

## Abstract

Oceanographers have an increasing responsibility to ensure that the outcomes of scientific research are conveyed to the policy-making sphere to achieve conservation and sustainable use of marine biodiversity. Zooplankton monitoring projects have helped to increase our understanding of the processes by which marine ecosystems respond to climate change and other environmental variations, ranging from regional to global scales, and its scientific value is recognized in the contexts of fisheries, biodiversity and global change studies. Nevertheless, zooplankton data have rarely been used at policy level for conservation and management of marine ecosystems services. One way that this can be pragmatically and effectively achieved is via the development of zooplankton indicators, which could for instance contribute to filling in gaps in the suite of global indicators to track progress against the Aichi Biodiversity Targets of the United Nations Strategic Plan for Biodiversity 2010–2020. This article begins by highlighting how under-represented the marine realm is within the current suite of global Aichi Target indicators. We then examine the potential to develop global indicators for relevant Aichi Targets, using existing zooplankton monitoring data, to address global biodiversity conservation challenges.

## VALUE OF ZOOPLANKTON MONITORING

Because of their small size and short lifecycle, zooplankton are sensitive to environmental stresses, which result in changes in zooplankton biomass and community structure. Such changes alter trophic linkages in marine food webs and affect the recruitment success of higher trophic levels. Zooplankton monitoring has been conducted in regional oceans worldwide from the early 20th century (Batten *et al.*, 2003) to the present (O’Brien *et al.*, 2017).

With the needs for better understanding of bottom-up control of variability fisheries resources, early zooplankton monitoring programs focused mainly on variability in biomass (Cushing, 1990; Reid *et al.*, 2003; McClellan *et al.*, 2014). Since the start of the Global Ocean Ecosystem Dynamics (GLOBEC) project in the early 1990s (Barange *et al.*, 2010) and through the follow-on Integrated Marine Biosphere Research (IMBeR) (Hofmann and IMBeR Scientific Steering Committee, 2016), it has been recognized that taxonomic breakdown, rather than mere biomass analysis, is required to understand the mechanisms linking the physical environment with higher trophic levels. Thus, variation in community structure and functional diversity became one of the main foci of zooplankton studies. Meanwhile, the Joint Global Ocean Flux Study (JGOFS) (Balino *et al.*, 2001) initiated a new phase of zooplankton research, focusing on their roles in the biogeochemical cycles including carbon transport to the deep ocean (Steinberg *et al.*, 2000; Schnetzer and Steinberg, 2002). Since the 2000s, the phenological changes (Edwards and Richardson, 2004; Chiba *et al.*, 2006; Mackas *et al.*, 2007; Richardson, 2008) and biogeographical shifts (Johns *et al.*, 2001; Beaugrand *et al.*, 2002; Batten and Walne, 2011; Keister *et al.*, 2011; Chiba *et al.*, 2015) in zooplankton communities responding to climatic forcing at various time-scales have been reported, and this knowledge has contributed to the studies of trends and future projections of climate change impacts on marine ecosystems (IPCC, 2014). More recently, in particular after the Census of Marine Life initiative (Costello *et al.*, 2010), marine biodiversity has been one of the key topics in zooplankton studies. Along with the expansion of study foci, the geographic range of studies has also expanded from regional to global. Quasi-global and global comparisons of the marine ecosystem variability have been conducted using zooplankton monitoring data (Rombouts *et al.*, 2010; Mackas *et al.*, 2012; Beaugrand *et al.*, 2014; O’Brien *et al.*, 2017), and sharing and long-term preservation of data are facilitated by the Ocean Biogeographic Information System (OBIS, http://www.iobis.org). To help with coordinating efforts, the Global Alliance of Continuous Plankton Recorder Survey (GACS), a global network of long-standing regional zooplankton monitoring programmes, was launched (Edwards *et al.*, 2011).

As the United Nations (UN) has designated 2021–2030 as the Decade of Ocean Science to achieve Sustainable Development Goal (SDG) 14: Conserve and sustainably use the oceans, seas and marine resources (https://en.unesco.org/ocean-decade), ocean scientists are required to be conscious of the societal benefits of their scientific outcome more than ever before. One way of effective communication between scientists and society is through the use of indicators, which represent scientific facts on environmental pressures, ecosystem states in ways that are more understandable ways for non-specialists (Brummitt *et al.*, 2017). Given the accumulated knowledge on zooplankton biology/ecology and the good temporal and geographical coverage of its monitoring efforts, many regional programmes have developed zooplankton indicators for assessment of various aspects of marine ecosystem services such as fisheries (Peterson and Burke, 2013) and ecosystem health (Racault *et al.*, 2014; Richardson *et al.*, 2015), and the usefulness of zooplankton indicators for their respective targets has been examined (Rombouts *et al.*, 2013; Setälä *et al.*, 2014; Uusitalo *et al.*, 2016; McQuatters-Gollop *et al.*, 2017; Jernberg *et al.*, 2017). Although the use of zooplankton indicators for better management options was recommended by scientists (Edwards *et al.*, 2010; McQuatters-Gollop *et al.*, 2017), they have rarely been used in the policy-making. This is a contrast to phytoplankton indicators, which have been applied for regional management policy of coastal eutrophication (OSPAR Commission, 2017) and environmental quality in the context of the Marine Strategy Framework Directive (McQuatters-Gollop *et al.*, 2015).

What can be done to help zooplankton indicators be used by policy? The international biodiversity conservation agenda established by the UN Convention on Biological Diversity (CBD) appears to provide an opportunity. Under the UN Strategic Plan for Biodiversity (2010–2020), a suite of global-scale indicators has been implemented to track progress in conservation and management of global biodiversity against the 20 so-called Aichi Biodiversity Targets (hereafter Aichi Targets) (https://www.cbd.int/sp/targets/). Although the Aichi Targets include various subjects relevant to marine environment and ecosystems, biological oceanographers including plankton biologists have rarely attempted to develop relevant the Aichi Target indicators. This is partly because Aichi Targets typically focus more on the terrestrial realm rather than the marine realm, and partly because zooplankton itself is not appealing as other charismatic groups in marine ecosystems such as sea birds, marine mammals and coral reefs. These owe to insufficient communication between oceanographers and the biodiversity conservation community, which may not realize the relevance of zooplankton to ecosystem health.

However, it is a fact that zooplankton supports a number of the Red List species in the oceans and the health of vulnerable marine ecosystems (Sims and Quayle, 1998; Jessopp *et al.*, 2013; McClellan *et al.*, 2014) both directly and indirectly.

This article aims to encourage zooplankton biologists to promote the use of zooplankton monitoring data for policy-making for biodiversity conservation and management through the development of the Aichi Target indicators, and also to urge the biodiversity conservation and oceanographic communities to strengthen their collaboration to enable the effective use of ocean observing information to reach their common fundamental goal: the sustainable use of marine biodiversity. In the following sections, we identify the marine relevance of the Aichi Targets and examine the potential of developing indicators using zooplankton data to fill the current gaps in the Aichi Target global indicator suites.

## AICHI BIODIVERSITY TARGETS FOR THE MARINE REALM

**Fig. 1.**
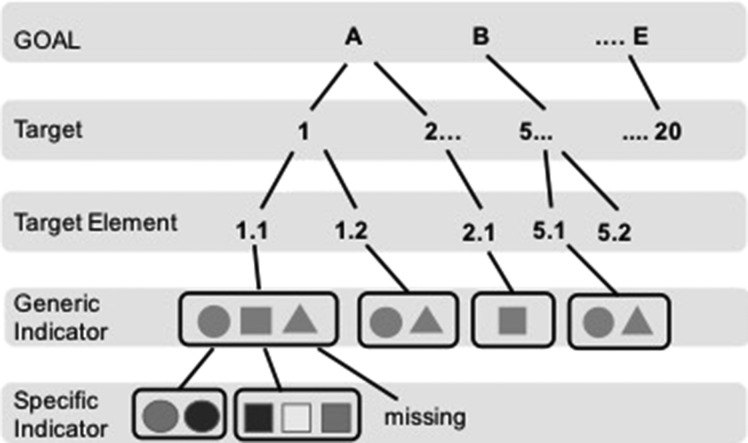
Schematic diagram of the hierarchical structure of the Aichi Biodiversity Targets and indicators.

The 20 Aichi Targets are categorized under the five goals (https://www.cbd.int/sp/targets/). In this article, we analyse Targets 5–16, which fall within Goals B: reduce pressures on biodiversity and promote sustainable use, Goal C: improve the status of biodiversity and Goal D: enhance the benefits to all from biodiversity and ecosystem services. Goal A (Targets 1–4) and Goal E (Targets 17–18) are focused on the response of society and policy rather than on environmental pressures and ecosystem states. Each target has its own “generic indicators”, which have matching “specific indicators” developed or proposed to monitor and assess the trend and achievement of the respective Aichi Targets at the global scale (Convention on Biological Diversity, 2016) (Fig. 1). For example, for Target 5, i.e. “By 2020, the rate of loss of all natural habitats, including forests, is at least halved and where feasible brought close to zero, and degradation and fragmentation is significantly reduced”, one of the generic indicators is “Trends in extent of natural habitats other than forest” and its specific indicator is “Wetland Extent (Dixon *et al.*, 2016)”.

The Biodiversity Indicators Partnership (BIP) curates the Aichi Targets indicators (Biodiversity Indicators Partnership, 2011). Given that some components of the Aichi Targets are still lacking global indicators (Mcowen *et al.*, 2016), the BIP has been coordinating and convening partner organizations, who develop specific indicators to fill the gaps in the indicator’s suites. Although all targets are more or less applicable to both terrestrial (including fresh water ecosystems) and marine ecosystems, the marine realm is under-represented, or at least its relevance is not clearly visible in the present set of specific indicators developed or proposed by the BIP partners.

**Fig. 2.**
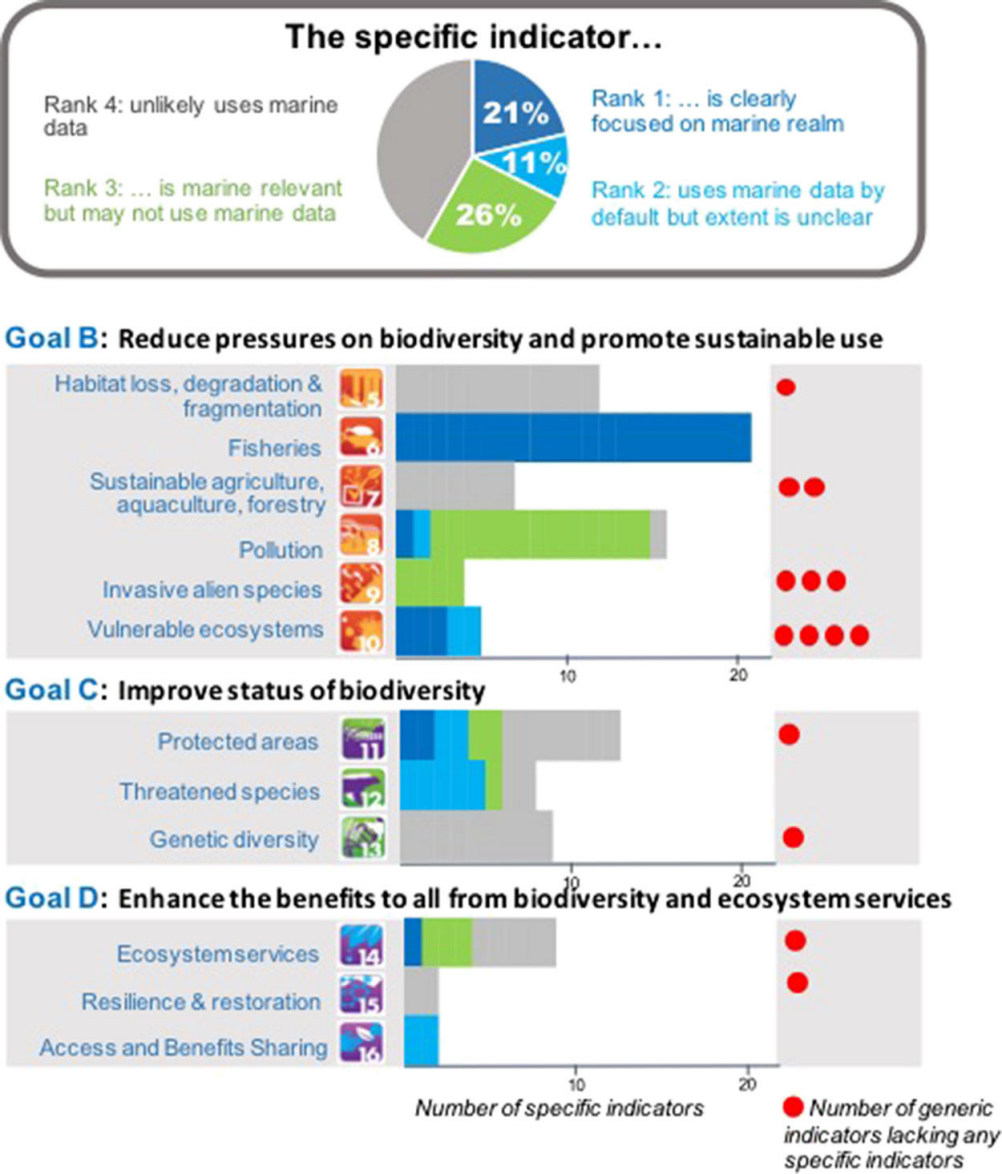
Number (bar) and ratio (pie) of the specific indicators developed or proposed for the Goals B, C and D of the Aichi Biodiversity Targets with ranking of their marine relevance. Red dots indicate the number of generic indicators that have no matching specific indicators, either terrestrial or marine (as of January 2017). See the indicator list (CBD/COP13): https://www.cbd.int/doc/decisions/cop-13/cop-13-dec-28-en.pdf.

To examine the under-representation of the marine realm in these indicators, we categorized the current specific indicators into four ranks depending on the extent of their marine relevance: Rank 1, clearly focuses on the marine realm (Fig. 2), e.g. “(fisheries) Catch certified by the Marine Stewardship Council (MSC, 2017)” for Target 6 on fisheries; Rank 2, marine data are included by default but their extent is unclear, e.g. Red List Index (Bubb *et al*., 2009) for Target 12 on threatened species; Rank 3, relevant to the marine realm but not clear if marine data are used, e.g. “Trends in global surplus of nitrogen” for Target 8 on pollution; and Rank 4, marine data are unlikely to be used or there is an exclusively terrestrial focus, e.g. “Number of plant genetic resources for food and agriculture surveyed/inventoried” for Target 13 on genetic diversity in socio-economically and culturally valuable species.

The results show that only 23 (21%) of the 108 specific indicators that were developed or proposed by the end of 2016 are clearly focused on the marine realm (Rank 1). There are no marine-relevant specific indicators at any levels for Targets 5, 7, 13 and 15, and their relevance is limited or unclear in Targets 8, 9 and 14 (Fig. 2). For Target 10 on multiple anthropogenic pressures and vulnerable ecosystems, some marine-relevant specific indicators have already been proposed, e.g. trend in proportion of live coral cover, and efforts are on-going to identify a partner organization responsible for producing and maintaining the indicator. However, four generic indicators of Target 10 still lack matching specific indicators (Fig. 2). One of those generic indicators, “Trends in extent and condition of vulnerable ecosystems (other than coral) impacted by climate change or ocean acidification”, is clearly relevant to the states of marine ecosystems; thus, its specific indicator(s) can be developed using marine biological observation data.

We examined the potential for development of marine-relevant specific indicators for the above Targets using the existing and developing observation networks and data sharing protocols already in place in the oceanographic community (Table I). Using a Driver-Pressure-State-Impact-Response (DPSIR) framework, the Biology and Ecosystem Panel of the Global Ocean Observation System (GOOS-BioEco) (Miloslavich *et al.*, 2018) has identified a set of Essential Ocean Variables (EOVs) to measure at global scale: phytoplankton biomass and diversity, zooplankton biomass and diversity, fish abundance and distribution, sea turtle/sea bird/marine mammal abundance and distribution, hard coral cover and composition, macroalgal canopy cover and composition, seagrass cover and composition and mangrove cover and composition. Conservation of marine biodiversity is one of the societal drivers framing the EOVs. As GOOS will urge the international ocean observing community to coordinate global observation network to implement respective EOVs, once the observation networks become fully functional, they would potentially provide the data needed to develop specific indicators to report the trend and state of marine ecosystems in terms of habitat loss (Target 5), invasive species (Target 9), vulnerable ecosystems (Target 10), ecosystem service (Target 14) and ecosystem resilience (Target 15) (Table I).

**Table I:** List of generic indicators of Aichi Targets that currently lack matching marine relevant specific indicators, and potential of development of marine relevant specific indicators using existing and/or planning ocean observation networks/initiatives

	Target	Generic indicator	Marine-related specific indicators possibly developed by..
**5**	By 2020, the rate of loss of all natural habitats, including forests, is at least halved and where feasible brought close to zero, and degradation and fragmentation is significantly reduced	Trends in extent of natural habitats other than forest Trends in degradation of forest and other natural habitats Trends in fragmentation of forest and other natural habitats Trends in extinction risk and populations of habitat specialist species in each major habitat type	using GOOS-Bio/Eco Panel Essential Ocean Variables, e.g. **live coral, seagrass, macroalgae canopy**, also data from current observation network on **mangrove, salt marsh, etc.**
**7**	By 2020, areas under agriculture, aquaculture and forestry are managed sustainably, ensuring conservation of biodiversity	Trends in proportion of production of aquaculture under sustainable practices	collating existing **aquaculture** data
**8**	By 2020, pollution, including from excess nutrients, has been brought to levels that are not detrimental to ecosystem function and biodiversity	Trends in pollutants Trends in ecosystems affected by pollution Trends in nutrient levels	…. coordination of coastal observation networks or regional programs on pollutants and nutrients
**9**	By 2020, invasive alien species and pathways are identified and prioritized, priority species are controlled or eradicated, and measures are in place to manage pathways to prevent their introduction and establishment.	Trends in identification and prioritization of invasive alien species Trends in the distribution and populations of invasive alien species Trends in impacts of invasive alien species on ecosystems	using GOOS-Bio/Eco Panel Essential Ocean Variables , e.g. **phytoplankton, zooplankton, fish, sea turtles/sea birds/marine mammals**
**10**	By 2015, the multiple anthropogenic pressures on coral reefs, and other vulnerable ecosystems impacted by climate change or ocean acidification are minimized, so as to maintain their integrity and functioning	Trends in extent and condition of other vulnerable ecosystems impacted by climate change or ocean acidification	using GOOS-Bio/Eco Panel Essential Ocean Variables , e.g. **phytoplankton, zooplankton, fish, sea turtles/sea birds/marine mammals**
		Trends in pressures on other vulnerable ecosystems impacted by climate change or ocean acidification	using data collated through GOOS-Physical panel, and GOOS-Biogeochemical Panel, and international carbon observation networks, e.g. GOA-ON, particularly for ocean acidification impacts
**13**	By 2020, the genetic diversity of cultivated plants and farmed and domesticated animals and of wild relatives, including other socio-economically as well as culturally valuable species, is maintained, and strategies have been developed and implemented for minimizing genetic erosion and safeguarding their genetic diversity	Trends in genetic diversity of socio-economically as well as culturally valuable species	using the best available genetic information of marine species, **e.g. fish, marine mammals, deep-sea benthos, coral reef species**
**14**	By 2020, ecosystems that provide essential services, including services related to water, and contribute to health, livelihoods and well-being, are restored and safeguarded, taking into account the needs of women, indigenous and local communities, and the poor and vulnerable	Trends in extinction risk and populations of species that provide essential services Trends in restoration of ecosystems that provide essential services	using GOOS-Bio/Eco Panel Essential Ocean Variables , **e.g. fish, sea turtles/sea birds/marine mammals, live coral, seagrass, macroalgae canopy**
**15**	By 2020, ecosystem resilience and the contribution of biodiversity to carbon stocks has been enhanced, through conservation and restoration, including restoration of at least 15% of degraded ecosystems, thereby contributing to climate change mitigation and adaptation and to combating desertification	Trends in ecosystem resilience	using GOOS-Bio/Eco Panel Essential Ocean Variables , **e.g. phytoplankton, zooplankton, fish, sea turtles/sea birds/marine mammals, live coral, seagrass, macroalgae canopy**
		Trends in carbon stocks within ecosystems	using data from remote-sensing and *in situ* observation of phytoplankton biomass and total particulate organic matters

Some of the generic indicators are for assessment of environmental “pressures” that impacts ecosystems, rather than the “state” of ecosystems. Because global observation systems are much advanced in terms of physical and biogeochemical variables, their observation networks could readily contribute to development of indicators to assess multiple environmental pressures on vulnerable marine ecosystems, e.g. specific indicator(s) could potentially be developed using chemical environmental data collated by the Global Ocean Acidification Observation Network (GOA-ON) (Newton *et al.*, 2014) to match the generic indicator “Trends in pressures on other vulnerable ecosystems impacted by climate change or ocean acidification” for Target 10. It is worth noting that “Average marine acidity (pH)” is designated as the official indicator for the United Nations Sustainable Development Goal 14.3: Minimize and address the impacts of ocean acidification, including through enhanced scientific cooperation at all levels.

## GLOBAL ZOOPLANKTON INDICATORS FOR THE AICHI TARGETS

With its existing long-term global observation efforts, zooplankton biomass and diversity were identified as one of the most mature EOVs of GOOS-BioEco in terms of readiness for observation at a global scale (Miloslavich *et al.*, 2018). We examine the strength of the global zooplankton data and zooplankton indicators, which have been proposed by various projects, against the criteria for the official global specific indicators for Aichi Targets. Among the criteria that BIP define are: (i) relevance and alignment to the respective target; (ii) good temporal coverage with at least five data points and the end data point no earlier than the year of 2010; (iii) good spatial coverage, ideally at the global scale; and (iv) scientific credibility of the indicator developed (Tittensor *et al.*, 2014). Here, we particularly consider the development of the specific indicators for the generic indicator of Target 10, “Trend in extent and condition of other (than coral reef) vulnerable ecosystems impacted by climate change of ocean acidification” and that of Target 15, “Trends in ecosystem resilience”.

Global zooplankton time-series metadata collated by the IOC-UNESCO International Group for Marine Ecological Time Series (IGMETS) report that nearly 200 regional projects have been conducting seasonal to annual observations for at least 5 years in the global ocean, and three-quarters of these have some level of taxonomic information, such as total copepod abundance (O’Brien, 2017) that meets the criteria of temporal and spatial scales. Existing zooplankton indicators, developed from its taxonomic and functional compositions for assessment of state and temporal trend of ecosystem health against various environmental pressures, have been published in peer-reviewed journals (Table II) and that meet the relevance and scientific credibility criteria for the Targets 10 and 15.

**Table II:** Description example zooplankton data which are potentially obtained thorough existing monitoring projects and will be useful for development of global indicators for Aichi Target 10 and 15

Variable type	What to Indicate & References to Support	Feasibility of Implementation
1. Total zooplankton abundance/biomass	Food quantity of higher trophic levels. Many (e.g. Brodeur, *et al.*, 1992; Cushing, 1995)	**Strength:** Data available for most of time-series, thus with great temporal and spatial coverage.
		**Challenge:** Cannot detect functional change and either negative and positive correlation between zooplankton biomass are observed, thus interpretations of state and trend are not robust.

2. States of target species/taxa. e.g. abundance of key stone species or functional type in the respective regional ecosystem.	Food quality of regionally important higher trophic level species, e.g. whales, salmon Deterioration of environment Invasive species Antarctic krill (Atkinson *et al.*, 2004; Constable *et al.*, 2016), Total copepods (Edwards *et al.*, 2002) *Calanus* spp. (Edwards *et al.*, 2011); *Neocalanus* spp. (Peterson and Burke, 2013) Jelly fish (Brotz *et al.*, 2012; Richardson *et al.*, 2015)	**Strength:** Effective for assessment in regions with specific ecosystems. Taxonomic analysis relatively easy.
		**Challenge:** Although not impossible, systematic integration of regionally specific information is needed to develop a global indicator, e.g. changes in functional types (grazer plankton, gelatinous plankton, etc.) against specific environmental pressures.

3. Size composition e,g. Copepod Community Size (*based on the female body size of each species) (Richardson *et al.* 2006)	Food quality of higher trophic levels, Biogeographical shifts (Richardson *et al.*, 2006; Chiba *et al.*, 2015)	**Strength:** What to indicate is relatively clear and applicable over various regions.
		**Challenge:** Need taxonomic analysis of all species, and literary information of average size of all species.

4. Community structure e.g. Principal Component value, NMDS score, etc.	Biodiversity, food quantity of higher trophic levels Biogeographical shifts (Beaugrand *et al.*, 2002, 2003; Beaugrand and Kirby, 2010; Keister *et al.*, 2011) Efficiency in carbon sequestration by biology Beaugrand *et al.* (2010)	**Strength:** Comprehensive analysis of ecosystem states.
		**Challenge:** Need taxonomic analysis of all species, and plausible explanation of what PC components indicate.

Table II summarizes the descriptions of four example zooplankton variables that could potentially be obtained at least at quasi-global scale through existing monitoring projects, and will be useful to develop global indicators of Aichi Targets 10 and 15: (i) total abundance/biomass, (ii) state of target species/taxa, (iii) size composition and (iv) community structure. Total abundance/biomass data are best available at global scale, but are not useful to understand functional changes in food web and ecosystems (Brodeur and Ware, 1992; Cushing, 1995). The states of target species (key stone species and hazardous species), e.g. Antarctic krill in the Antarctic Ocean (Atkinson *et al.*, 2004; Constable *et al.*, 2016) and Jellyfish (Brotz *et al.*, 2012), indicate changes in the regional ecosystem functioning. The relative abundance of dominant species, e.g. *Calanus* spp. in the North Atlantic (Edwards *et al.*, 2011) and *Neocalanus* spp. in the North Pacific (Peterson and Burke, 2013) indicates the food quality for target fisheries resources. The average body size of the zooplankton community indicates the shifts in the major zooplankton functional types and food quality for planktivorous fish, birds and mammals (Richardson *et al.*, 2006) and biogeographical shifts of ecosystems (Chiba *et al.*, 2015). Community structure information obtained by multivariate analysis methods indicates changes in the ecosystem structure, e.g. both functional and species diversity, in a comprehensive manner (Beaugrand *et al.*, 2002, 2003; Beaugrand and Kirby, 2010; Keister *et al.*, 2011) and may indicate the efficiency of biological carbon sequestration (Beaugrand *et al.*, 2010). Since information of the zooplankton indicators 2, 3 and 4 indicates ecosystem stability against environmental pressures over the time, a marine ecosystem resilience index for the Target 15 can be developed from those variables via coupling with physical and biogeochemical data.

One of the major issues remaining for development of global indicators from zooplankton variables is the compatibility of data that are collected, processed and analysed using project-specific sampling gears and analytical methods. There is an increasing demand to develop new sensor technology to enable autonomous measurement of taxonomic or functional level information of zooplankton data (Le Bourg *et al.*, 2014; Watson, 2018). Once these technologies become matured and available to the international observation community, zooplankton biodiversity information can be collected with standardized methods using various existing observation platforms such as buoys, gliders and moorings. A recent effort to automatize the collection of zooplankton data is being discussed by a Scientific Committee in Oceanic Research (SCOR) Working Group (Boss *et al.*, 2018). This group, Integration of Plankton-Observing Sensor Systems to Existing Global Sampling Programs (P-OBS), is focused on identifying best practices (technologies and sampling) to incorporate plankton observations into global observing platforms such as GO-SHIP and OceanSites and in the challenge of lack of standardization and protocols to obtain trustable, quality controlled and open access data (Boss *et al.*, 2018). However, it will still take a long time for these systems to be operated in a cost-effective manner with good temporal and spatial coverages, also there will be challenges in calibrating these data against the existing time series so that hind-casting is possible.

In this sense, GACS, established in 2011 (Edwards *et al.*, 2011), is currently the most robust zooplankton monitoring network that could contribute to the implementation of zooplankton EOVs and delivery of Aichi Target indicators. Using the Continuous Plankton Recorder (CPR) system developed by the Sir Alister Hardy Foundation for Ocean Science (SAHFOS) (Reid *et al.*, 2003), the participant organizations from nine countries apply a well-standardized protocol for sampling and analysis (Batten *et al.*, 2003). Owing to the nature of observation using Ships of Opportunity, CPR data include information from the high seas and transboundary regions, which will be highly valuable for international policy-making processes aiming to conserve Biodiversity in the areas Beyond National Jurisdiction (BBNJ), where biodiversity information is much more limited than in coastal areas and waters within national Exclusive Economic Zones (Rogers *et al.*, 2014).

**Fig. 3.**
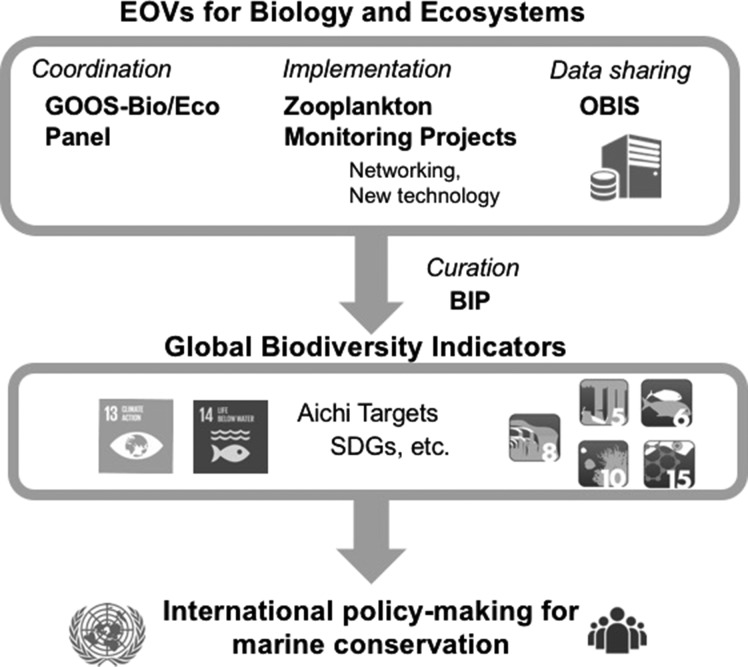
Workflow from establishment and implementation of the GOOS-Bio/Eco zooplankton EOVs to development of global biodiversity indicators for the UN-related projects and contribution to International marine conservation policy, with the roles of the respective organizations/groups involved.

Although the GACS network is already quasi-global, there are some spatial gaps, especially in lower latitude regions, though the special coverage is comparatively excellent in contrast to most other biological compartments apart from phytoplankton. For implementation of the zooplankton EOVs at a fully global scale, it is necessary to seek the best practice for interoperability of observation and integration of data among GACS and other monitoring projects. Even though thorough standardization of the sampling methodology among those projects is unrealistic as each project is designed for its own scientific foci and societal demands, global comparison of regional time-series data will still be possible by applying and/or developing similar methods such as “Mackas method” (O’Brien *et al.*, 2017), which extracts trends of various time-series by calculating the slope of annual anomalies. In summary, along with the establishment of a sustained, multidisciplinary global observation network to implement GOOS zooplankton EOVs, the zooplankton science community is urged to become BIP partners and to establish robust protocols to report the respective Aichi Target global indicators to address global biodiversity conservation challenges (Fig. 3).

## CONCLUDING REMARKS

Monitoring of zooplankton and diverse marine ecosystem can contribute more proactively to international biodiversity conservation frameworks. Observation networks and data sharing protocols among existing regional zooplankton monitoring programmes should evolve rapidly in the next decade, which would ensure generation of quality-controlled data on a global scale to increase representation of the marine realm in the Aichi Biodiversity Target indicator suites. With the initial due date of 2020 for achieving the Aichi Targets, the global biodiversity conservation initiative is currently designing its strategic plan beyond 2020 in which linking the Aichi Targets and SDGs are recommended (Convention on Biological Diversity, 2017). Zooplankton biologists and oceanographers are encouraged to get involved in their planning process to ensure effective utilization of their data in decision making on the ecosystem health of the one ocean.
